# The Performance of the Four Anaerobic Blood Culture Bottles BacT/ALERT-FN, -FN Plus, BACTEC-Plus and -Lytic in Detection of Anaerobic Bacteria and Identification by Direct MALDI-TOF MS

**DOI:** 10.1371/journal.pone.0142398

**Published:** 2015-11-10

**Authors:** Mohammed Almuhayawi, Osman Altun, Adam Dilshad Abdulmajeed, Måns Ullberg, Volkan Özenci

**Affiliations:** 1 Division of Clinical Microbiology F 72, Karolinska Institutet, Karolinska University Hospital, Huddinge, SE 141 86, Stockholm, Sweden; 2 Department of Microbiology, King Abdul-Aziz University, Jeddah, Saudi Arabia; The University of Hong Kong, HONG KONG

## Abstract

Detection and identification of anaerobic bacteria in blood cultures (BC) is a well-recognized challenge in clinical microbiology. We studied 100 clinical anaerobic BC isolates to evaluate the performance of BacT/ALERT-FN, -FN Plus (BioMérieux), BACTEC-Plus and -Lytic (Becton Dickinson BioSciences) BC bottles in detection and time to detection (TTD) of anaerobic bacteria. BACTEC Lytic had higher detection rate (94/100, 94%) than BacT/ALERT FN Plus (80/100, 80%) (p<0.01) in the studied material. There was no significant difference in detection of anaerobic bacteria among the remaining bottle types. The 67 anaerobic bacteria that signalled positive in all four bottle types were analyzed to compare the time to detection (TTD) and isolates were directly identified by MALDI-TOF MS. There was a significant difference in TTD among the four bottle types (p<0.0001). The shortest median TTD was 18 h in BACTEC Lytic followed by BacT/ALERT FN (23.5 h), BACTEC Plus (27 h) and finally BacT/ALERT FN Plus (38 h) bottles. In contrast, MALDI-TOF MS performed similarly in all bottle types with accurate identification in 51/67 (76%) BacT/ALERT FN, 51/67 (76%) BacT/ALERT FN Plus, 53/67 (79%) BACTEC Plus and 50/67 (75%) BACTEC Lytic bottles. In conclusion, BACTEC Lytic bottles have significantly better detection rates and shorter TTD compared to the three other bottle types. The anaerobic BC bottles are equally suitable for direct MALDI-TOF MS for rapid and reliable identification of common anaerobic bacteria. Further clinical studies are warranted to investigate the performance of anaerobic BC bottles in detection of anaerobic bacteria and identification by direct MALDI-TOF MS.

## Introduction

Anaerobic bacteraemia is associated with a mortality rate of 15–30% and accounts for 1–17% of all positive blood cultures (BC) depending on the clinical setting [1]. Early recognition and appropriate treatment of anaerobic bacteraemia are decisive in order to improve the prognosis [2,3]. It was shown that antimicrobial therapy was altered for 56% of the patients with a positive anaerobic blood culture [4].

Detection and identification of anaerobic bacteria in BC is a well-recognized challenge in clinical microbiology. This may be explained by the fact that these microorganisms are typically fastidious, slow growing and difficult to culture. However, the developments of automated BC systems and anaerobic BC bottles have improved the detection of these microorganisms [3,5,6].

Prospective clinical studies have been crucial to evaluate the performance of the BC bottles. However, appropriate comparison in detection of rare microorganisms such as anaerobic bacteria in prospective clinical studies is tremendously challenging [7–10]. The most common BC systems that are used worldwide are BD BACTEC (Becton Dickinson Instrument Systems, Sparks, MD) and BACT/ALERT^®^ 3D (bioMérieux, Marcy l'Etoile, France). The four most common types of anaerobic BC bottles used in these two systems are BacT/ALERT-FN, -FN Plus (BioMérieux), BACTEC-Plus and -Lytic (Becton Dickinson). The comparison of these anaerobic bottles for detection and time to detection (TTD) of anaerobic bacteria has not been studied previously.

Matrix-assisted laser desorption/ionization time-of-flight mass spectrometry (MALDI-TOF MS) is a simple approach to identify bacteria and yeast in minutes directly from colonies grown on agar plates [11,12]. However, the published data on identification of anaerobic bacteria directly from BC bottles by MALDI-TOF MS are based on limited numbers of isolates [13,14].

The aim of the present study was to (i) compare the performance of BacT/ALERT-FN, -FN Plus (BioMérieux), BACTEC-Plus and -Lytic (Becton Dickinson) BC bottles in detection and (ii) TTD of anaerobic bacteria, and to (iii) evaluate the performance of direct MALDI-TOF MS in identification of anaerobic bacteria directly from positive bottles.

## Materials and Methods

### Study design and material

The study was performed at Karolinska University Laboratory in Huddinge, Sweden, which serves the southern part of the greater Stockholm area and surrounding cities and suburbs. The laboratory receives blood culture specimens from three tertiary-care hospitals: Karolinska University Hospital in Huddinge, Stockholm, South General Hospital, Stockholm, and Södertälje Hospital in Södertälje, with a total of 1,569 patient beds. Clinical anaerobic bacteria isolates used in the study were collected during the period of 2010–2012 from positive BC at Karolinska University Hospital, Huddinge, Sweden and stored at -70°C. The present species distribution reflects the clinical distribution of anaerobic bacteria isolated from blood cultures in our centre.

### Conventional methods

Gram stains were done directly from positive blood culture bottles. According to the results of the staining, specimens from the positive bottles were subcultured onto relevant agar plates. The microorganisms grown on the agar plates were identified by Vitek2 XL (bioMérieux, Marcy l’Etoile, France), by Bruker MALDI-TOF MS (Bruker Daltonics, Bremen, Germany), by growth characteristics on selective agar plates and by a panel of validated desktop spot tests, including catalase, and indole spot. The isolates (n = 100) included in the study constituted of *Bacteroides fragilis* (n = 35), *Clostridium perfringens* (13), *Bacteroides thetaiotaomicron* (12), *Bacteroides ovatus* (4), *Fusobacterium nucleatum* (4), *Eggerthella lenta* (4), *Bacteroides vulgates* (3), *Clostridium ramosum* (3), *Veillonella parvula* (2), *Clostridium cadaveris* (2), *Bifdobacterum breve* (2), *Propinibacterium acnes* (2), *Clostridium septicum* (2), *Prevotella buccae* (2), *Veillonella atypical* (1), *Alistipes fingoldii* (1), *Fusobacterium mortiferum* (1), *Clostridium tertium* (1), *Clostridium clostridioforme* (1), *Clostridium hathewayi* (1), *Clostridium innocuum* (1), *Parabacteroides goldsteinii* (1), *Propionibacterium* species (1) and *Lactobacillus* species (1). All bacterial species included in the study were present in the Bruker Biotyper 3.1 software and library (version 4613, Bruker Daltonics) used for spectrum analysis.

### Blood culture system and bottles

Four different blood culture bottles from 2 different blood culture systems were included in the study. The BacT/ALERT FN and BacT/ALERT FN Plus bottles were incubated in the BacT/Alert 3D (bioMérieux, Durham, NC, USA) and the BACTEC Plus and BACTEC Lytic bottles were incubated in the the BD BACTEC FX (BD Diagnostic Systems, Sparks, MD, USA) automated blood culture systems. BacT/ALERT FN blood culture bottles contain 32ml of complex media and 8ml of a charcoal suspension with an average density of 1.0215 g/mL. BacT/ALERT FN Plus blood culture bottles include 40 ml of complex medium and 1.6 g adsorbent polymeric beads. The BACTEC Plus anaerobic culture bottles contain 25 ml complex media and 16.0% nonionic adsorbing- and 1.0% cationic exchange-resin. The BACTEC Lytic Anaerobic/F culture bottles contain 40 ml of complex medium and no charcoal suspension or adsorbent beads.

### Simulated blood cultures

Spiked cultures were prepared by sub-culturing anaerobes onto anaerobic agar medium and incubate them in anaerobic jars at 35°C for 48 h. Colonies from agar plates were then resuspended in phosphate buffer solution to 0.5 McFarland (1.5 x 10^8^ CFU/ml) and diluted 1:100 two times to a final concentration of approximately 1.5 x 10^4^ CFU / ml. Fifty microliters from the this last suspension (ca 750 CFU) was inoculated in each type of anaerobic BC bottle, i.e. BacT/AlertALERT-FN, -FN Plus (bioMérieux, Marcy l'Etoile, France), BACTEC- Plus and -Lytic (Becton Dickinson Instrument Systems, Sparks, MD) anaerobic blood culture vial. Inoculum densities were verified by culturing 50 μl of the final suspensions on each of three blood agar plates that were incubated in anaerobic atmosphere for 48 h. Inoculation size was based on quantitative aspects of septicaemia as described previously (Yagupsky 1990). The bottles were inoculated with five ml hHorse blood and incubated in the respective blood cultureBC system BacT/ALERTALERT 3D (bioMérieux) and BACTEC 9240 (BD) until signalling for positivity or for a maximum of five days. Bottles that did not signal positive at the end of five days were sub-cultured on agar plates (48 h in anaerobic jars at 35°C) for verification of false/true negativity. Positive blood cultures were used to evaluate MALDI-TOF MS (Bruker Daltonic, Germany) in identification of anaerobic bacteria directly from blood cultureBC bottles. Discrepant results between conventional identification methods and MALDI-TOF MS were analyzed by partial 16S rRNA gene sequencing.

### MALDI-TOF MS

Five ml broth from positive bottles was centrifuged at 180 relative centrifugal force (rcf) for 10 min. Then, 1.5 ml of the supernatant was transferred to an Eppendorf tube and centrifuged at high speed (24,800 rcf) for 1 min. The pellet was washed twice by discarding the supernatant, adding 1 ml deionised water and centrifugation of the specimen at high speed. Three microliters of organic acid (OS) [50% Acetonitrile (Fisher, Chemicals UK), 47.5% non-ionized water, 2.5% trifluoroacetic acid (TFA) v/v] and 20 μl TFA (Merck KGaA, Darmstadt, Germany) was added to the pellet. A thin layer of the pellet/OS mix was applied with a wooden applicator to the MALDI-TOF target plate (MSP 96 target, Bruker Daltonics, Bremen, Germany). One microliter formic acid 70% (Merck KGaA, Darmstadt, Germany) was added and left to dry before applying one microliter of matrix alph-cyano-4-hydroxycinnamic acid (Bruker Daltonics). MALDI-TOF MS (Bruker Daltonics, Germany) analysis was performed with the Bruker Biotyper 3.1 software and library (version 4613, Bruker Daltonics) at the mass spectra ranging from 2,000 to 20,000 Daltons.

The recommended score values by the manufacturer are >1.7 for genus level and >2.0 for species level and are based on identification of microorganisms from solid medium. However, identification of microorganisms from liquid medium such as BC broth is associated with non-bacterial background peaks (human proteins, components of broth and charcoal) [12,15,16], This creates a lower quality of the spectrum and subsequently lower score values. Wuppenhorst et. al., (2012) showed that modified score criteria were suitable in identification of microorganisms directly from BC bottles and provided reliable identification of microorganisms [15]. Accordingly, in the present study, species level identification was considered reliable for any log score over 1.7. Scores ranging between ≥1.4 and ≤1.7 were considered reliable for genus identification if the first three matched patterns were the same. Scores <1.4 were interpreted as no identification and were based on analyzing 10 negative BC bottles with direct MALDI-TOF (data not shown). The MALDI-TOF scores of <1.4 for negative bottles were in line with previously published study [15].

### Partial 16S rRNA gene sequencing

Partial sequencing of the 16S rRNA gene including the hypervariable regions V3 and V4 was performed for two isolates with discrepant results. Bacterial DNA was extracted using the automated Biorobot M48 system (Qiagen, Hilden, Germany), according to the manufacturer's instructions. PCR amplification was performed by adding 3 μl of the extract to a master mix containing 10 μM of each primer (5´-CGGCCCAGACTCCTACGGGAGGCAGCA-3´ and 5´-GCGTGGACTACCAGGGTATCTAATCC-3´) together with 25 μl HotStarTaq master mix (Qiagen) to give a final volume of 50 μl. After initial denaturation, the thermocycling parameters were 32 cycles at 94°C for 30s, 56°C for 30s, and 72°C for 1 min, followed by a final extension at 72°C for 7 min. The PCR products were purified using chemical purification (PE Applied Biosystems) according to the manufacturer's instructions. Sequencing of both strands was carried out using an ABI Prism BigDye Terminator v3.1 cycle sequencing kit (Applied Biosystems, Foster City, CA) with a GeneAmp 9700 thermocycler (Applied Biosystems). Sequencing primers used in the two reactions were 5′-AGAGTTTGATCMTGGCTCAG-3′ and 5′-GWATTACCGCGGCKGCTG-3′, 1 μM each. The sequence cycling products were analyzed by capillary electrophoresis and fluorescence detection with an Applied Biosystems ABI 3100 genetic analyzer. The fluorescence data were analyzed with the SeqScape Software program (version 4.5; Gene Codes Corporation, Ann Arbor, MI). BLAST search showed 100% nucleotide identity to previously registered sequences of the 16S rRNA gene of *Alistipes fingoldii* (330/330 bases) and *Parabacteroides goldsteinii* (330/330 bases).

### Statistical analysis

The positivity rates for the four bottle types were compared using the Chi-square test. Fisher’s exact test was used to compare two different blood culture bottles. The TTD was compared by the Wilcoxon matched-pairs signed-rank test. Values of p<0.05 were considered to be statistically significant.

## Results

### Detection of growth

In total, 400 anaerobic BC bottles were included in the study, comprising four sets of different BC bottles inoculated with the same 100 anaerobic bacteria. During 5 days incubation, 89/100 (89%) BacT/ALERT FN, 80/100 (80%) BacT/ALERT FN Plus, 85/100 (85%) BACTEC Plus and 94/100 (94%) BACTEC Lytic bottles signalled positive in the BC systems. There was significant difference in detection of anaerobic bacteria among the four bottle types studied (p<0.05). BACTEC Lytic had significantly higher detection rate than BacT/ALERT FN Plus (p<0.01). There was no significant difference in detection of anaerobic bacteria among the remaining bottle types.

Table 1 depicts the growth characteristics of the remaining 33 anaerobic isolates that did not signal positive in all four types of bottles. The number of isolates per species was relatively small to draw conclusions regarding the detection of specific bacteria. However, trends in detection of certain bacterial species were observed among the bottles types. BacT/ALERT FN detected all 4/4 *E*. *lenta* while BACTEC Lytic did not detect any of the four *E*. *lenta*. Finally, BACTEC Lytic was the only BC bottle that detected growth of the two *P*. *acnes* included in the study.

**Table 1:** Detection and time to detection of anaerobic isolates that did not grow in all four blood culture bottles.

Microorganism (n = 33)	BacT/ALERT		BACTEC	
	FN (h)(No. NG = 11)	FN Plus (h)(No. NG = 20)	Plus (h)(No. NG = 15)	Lytic (h)(No. NG = 6)
*Alistipes finegoldii*	49.0	NG*	NG*	74.0
*Bacteroides fragilis*	23.8	69.1	NG*	22.0
*B*. *fragilis*	28.3	NG*	30.4	18.0
*B*. *fragilis*	25.0	51.0	NG*	18.1
*B*. *fragilis*	NG*	44.2	35.1	17.1
*B*. *fragilis*	23.0	NG*	29.0	22.0
*B*. *fragilis*	22.0	40.0	NG*	16.0
*B*. *fragilis*	24.5	NG*	23.2	17.1
*B*. *ovatus*	NG*	NG*	NG*	27.4
*B*. *thetaiotaomicron*	30.0	44.6	NG*	NG*
*B*. *thetaiotaomicron*	NG*	NG*	45.2	22.1
*B*. *thetaiotaomicron*	NG*	NG*	45.3	26.0
*B*. *thetaiotaomicron*	15.8	NG*	34.2	14.1
*B*. *thetaiotaomicron*	27.1	NG*	47.5	27.4
*B*. *breve*	40.1	NG*	68.2	21.3
*B*. *breve*	NG*	73.0	78.6	26.2
*Clostridium cadaveris*	18.0	NG*	24.1	24.5
*C*. *clostridioforme*	50.0	NG*	49.6	33.4
*C*. *perfringens*	16.0	21.0	NG*	NG*
*C*. *perfringens*	28.0	NG*	24.0	9.0
*C*. *perfringens*	13.0	NG*	11.6	8.6
*C*. *septicum*	NG*	17.0	NG*	12.0
*Eggerthella lenta*	29.0	39.8	38.3	NG*
*E*. *lenta*	32.6	NG*	42.4	NG*
*E*. *lenta*	29.8	NG*	NG*	NG*
*E*. *lenta*	26.0	45.0	NG*	NG*
*Fusobacterium ucleatum*	33.1	NG*	NG*	43.4
*F*. *nucleatum*	NG*	NG*	72.0	45.0
*Prevotella buccae*	NG*	108.0	72.0	36.0
*P*. *buccae*	19.0	22.0	NG*	75.0
*Propionibacterium acnes*	NG*	NG*	NG*	119.2
*P*. *acnes*	NG*	NG*	NG*	97.0
*P*. *species*	NG*	111.8	NG*	76.4

*NG (no growth) after 5 days incubation in the blood culture system.

Sub-culture of negative BC bottles detected six false negative bottles (i.e. growth was observed on agar plates after sub-cultures from negative bottles at the end of five days of incubation). These were four BACTEC Plus bottles (two *P*. *acnes*, one of each *Proprinobacterium* species and *P*. *buccae*) and one of each BacT/ALERT FN Plus (*P*. *acnes*) and BACTEC Lytic (*E*. *lenta*).

The 67 isolates (47 Gram-negative and 20 Gram-positive) that signalled positive in all four bottles types were used to compare the TTD and the performance of MALDi-TOF MS among bottle types. These isolates were *B*. *fragilis* (n = 28), *C*. *perfringens* (10), *B*. *thetaiotaomicron* (7), *B*. *vulgates* (3), *B*. *ovatus* (3), *C*. *ramosum* (3), *V*. *parvula* (2), *F*. *nucleatum* (2), *C*. *cadaveris* (1), *C*. *tertium* (1), *P*. *goldsteinii* (1), *F*. *mortiferum* (1), *C*. *innocuum* (1), *C*. *hathewayi* (1), *C*. *septicum* (1), *V*. *atypical* (1) and *Lactobacillus* species (1).

### The time to detection

**Fig 1:**
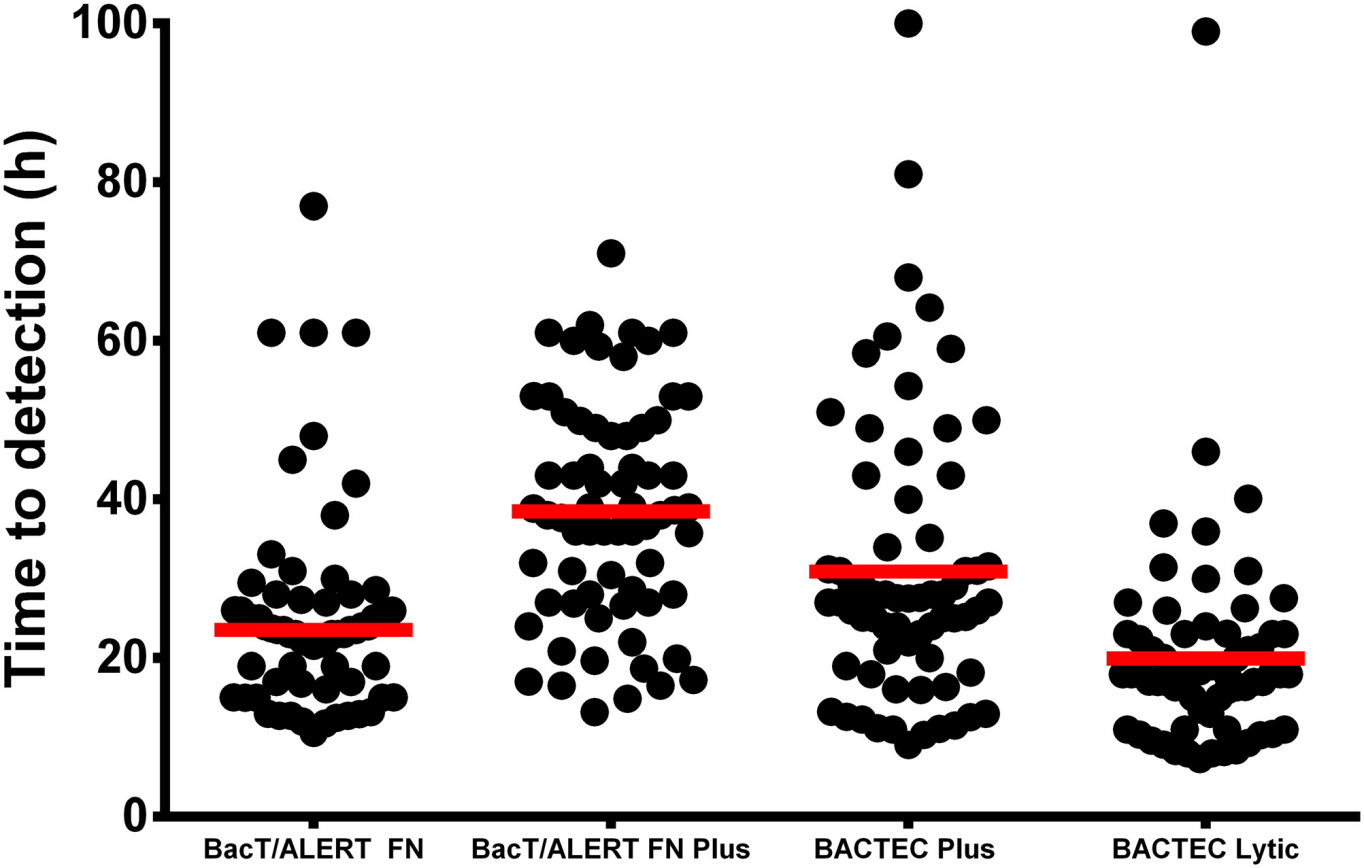
Time to detection (TTD) of the 67 anaerobic bacteria isolates that grew in all four types of anaerobic blood culture bottles. Each dot represents one blood culture bottle. Bars represent median TTD.

The shortest median TTD was 18 h in BACTEC Lytic followed by BacT/ALERT FN (23.5 h), BACTEC Plus (27 h) and finally BacT/ALERT FN Plus (38 h) bottles (Fig 1). There was a significant difference in the median TTD between the bottles types (p<0.0001 for all three comparisons). The TTD was significantly shorter in BACTEC Lytic compared to BacT/ALERT FN, BacT/ALERT FN Plus and BACTEC Plus bottles (p<0.0001 for all three comparisons). Similarly BacT/ALERT FN had shorter TTD than BACTEC Plus and BacT/ALERT FN Plus bottles (p<0.001 and p< 0.0001 respectively). BACTEC Plus had shorter TTD than BacT/ALERT FN Plus bottles (p<0.0001).

### MALDI-TOF MS

Overall, accurate identification was achieved in 51/67 (76%) from BacT/ALERT FN, 51/67 (76%) from BacT/ALERT FN Plus, 53/67 (79%) from BACTEC Plus and 50/67 (75%) from BACTEC Lytic bottles (Table 2). There was no difference in identification of anaerobic bacteria by MALDI-TOF MS among the four blood culture bottles included in the study.

**Table 2:** Identification of anaerobic bacteria directly from blood culture bottles by MALDI-TOF MS.

Microorganism	(n)	ID From all four bottles (n)	BacT/Alert (n)		BACTEC (n)	
			FN	FN Plus	Plus	Lytic
**Gram negative**	**47**	**33**	**37 (79%)**	**38 (81%)**	**37 (79%)**	**39 (83%)**
*Bacteroides fragilis*	28	23	24	24	23	25
*B*. *thetaiotaomicron*	7	6	7	6	7	7
*B*. *ovatus*	3	2	2	2	2	2
*B*. *vulgatus*	3	2	2	3	3	2
*F*. *nucleatum*	2	0	0	0	1	1
*V*. *parvula*	2	0	2	2	0	2
*V*. *atypica*	1	0	0	1	1	0
*Fusobacterium mortiferum*	1	0	0	0	0	0
**Gram positive**	**20**	**9**	**14 (70%)**	**13 (65%)**	**16 (80%)**	**11 (55%)**
*Clostridium perfringens*	10	6	8	6	8	6
*C*. *ramosum*	3	0	3	1	2	0
*C*. *cadaveris*	1	0	0	1	1	1
*C*. *septicum*	1	1	1	1	1	1
*C*. *tertium*	1	1	1	1	1	1
*C*. *innocuum*	1	0	0	1	1	1
*C*. *hathewayi*	1	0	0	1	1	0
*Parabacteroides goldsteinii*	1	1	1	1	1	1
Lactobacillus sp.	1	0	0	0	0	0
**Total (%)**	**67**	**42/67 (63%)**	**51/67 (76%)**	**51 (76%)**	**53 (79%)**	**50 (75%)**

Table 3 depicts the MALDI-TOF MS score distribution for the isolates identified directly from the blood culture bottles. No difference was observed in numbers of isolates identified with high (>2.0) MALDI-TOF scores among the four blood culture bottles. Only two BACTEC Lytic (3%) and one BACTEC Plus (2%) samples had low scores with correct identification (Table 3). Interestingly, all three isolates with low scores had MALDI-TOF MS scores between 1.6 and 1.69.

**Table 3:** MALDI-TOF MS identification rate in 67 isolates that grew in all 4 anaerobic blood culture bottles.

MALDI-TOF MS Score	BacT/ALERT n (%)		BACTEC n (%)	
	FN	FN Plus	Plus	Lytic
**>2** *	38 (57%)	36 (54%)	43 (64%)	34 (51%)
**1.9–1.7** *	13 (19%)	15 (22%)	9 (13%)	14 21%)
**1.69–1.4** **			1 (2%)	2 (3%)
**ID**	51 (76%)	51 (76%)	53 (79%)	50 (75%)
**No ID**	16 (24%)	16 (24%)	14 (21%)	17 25%)

* The scores 1.7-1-9 and >2 were considered as species level identification.

** Scores ranging between ≥1.4 and ≤1.7 were considered reliable for genus identification if the first three matched patterns were the same.

There were only two discrepant results between Vitek2 and MALDI-TOF MS results. Vitek2 identified *A*. *fingoldii* as *Prevotella* species and *P*. *goldsteinii* as *B*. *fragilis*. Partial 16S rRNA gene sequencing was performed on both samples and confirmed MALDI-TOF MS results.

## Discussion

Rapid detection and identification of anaerobic bacteria in BC is crucial for appropriate antimicrobial therapy [4,17]. The BC are considered as the “gold standard” for detecting bacteremia. The present study analyzed the performance of four different anaerobic BC bottles in detection and rapid identification of anaerobic bacteria.

Detection of anaerobic bacteria remains challenging due to the fastidious nature of these microorganisms. In the present study, BACTEC Lytic had significantly higher detection rate than BacT/ALERT FN Plus (p<0.01). There was no significant difference in detection of anaerobic bacteria among the other BC bottles. The underlying reason for the present difference between BACTEC Lytic and BacT/ALERT FN Plus is unknown. It is possible to suggest that the content of the BC bottles, the microorganisms tested and the studied material might be confounding factors for the present finding.

Rapid detection of growth in BC is clinically relevant as this influences initiation of antibacterial therapy early in the disease process. The performance of the bottle types in terms of TTD was measured for 67 clinical isolates that signalled positive in all four bottle types. Interestingly, we found a significant difference in the median TTD between the four bottle types (p<0.0001). BACTEC Lytic bottles had the shortest median TTD of 18 h, followed by BacT/ALERT FN (23.5 h), BACTEC Plus (27 h), and BacT/ALERT FN Plus (38 h) (Fig 1). The significant differences in TTD among the bottles types observed in the present study might have clinical implications including a later initiation of appropriate antimicrobial therapy [18].

Prompt identification in combination with local surveillance of antimicrobial susceptibility patterns may allow earlier initiation of appropriate antimicrobial therapy [19]. The use of MALDI-TOF MS as a rapid identification method for aerobic bacteria and yeasts directly from positive BC bottles has been widely established in microbiology laboratories [15,20–22]. This approach provides an opportunity to bypass sub-culturing of BC by identifying microorganisms directly from bottles. However, reliable MALDI-TOF MS identification of anaerobic bacteria from BC is done by first sub-culturing the broth on anaerobic plates for 48 h and then analyzing colonies from the anaerobic plates [23]. Hitherto published studies have shown promising results of identifying anaerobic bacteria directly from positive BC with MALDI-TOF MS [22,24]. However these studies included low number of BC bottles and few anaerobic species and further studies were warranted [25]. We investigated the performance of direct MALDI-TOF MS in four different simulated BC bottles. In order to make appropriate comparison between the four bottle types, only the 67/100 (67%) isolates that were positive in all four bottle types were statistically analyzed. Interestingly, direct MALDI-TOF MS performed similarly in all bottle types with accurate identification of at least 50/67 (75%) isolates including BacT/ALERT FN bottles that contain charcoal. In contrast, previous investigators have reported that charcoal containing bottles have a significant negative effect on direct MALDI-TOF MS [26]. In line with the present study, Wuppenhorst et. al. (2012) reported that the performance of direct MALDI-TOF MS is not significantly affected by charcoal [15]. The disparity between the studies could probably be explained by the use of different sample preparations before MALDI-TOF analysis.

Two isolates in this study highlighted the impressive width of the MALDI-TOF MS database. MALDI-TOF MS could identify *A*. *finegoldii* and *P*. *goldsteinii*, two very rare causes of bloodstream infections, directly from BC bottles. Repetitive identification from agar plates by MALDI-TOF MS gave the same results. Later, 16S rRNA gene sequencing confirmed the MALDI-TOF MS results. To our knowledge only two reports have showed *A*. *finegoldii* and *P*. *goldsteinii* as causative pathogens for bacteraemia [27,28]. Interestingly, conventional identification by Vitek2 XL (bioMérieux, France) identified *P*. *goldsteinii* as *B*. *fragilis* and *A*. *finegoldii* as *Prevotella* species. The underlying reason for misidentification by Vitek2 is that none of the two isolates are included in the Vitek2 ANC card panel.

There are possible limitations with conducting a study on simulated BC. The clinical BC might include parameters that are difficult to appropriately reproduce. These include variable composition of blood cells, antimicrobial content and transport time of BC bottles that are normally observed in clinical BC. However, appropriate comparison of the BC bottles in detection of rare microorganisms such as anaerobic bacteria in prospective clinical studies would be tremendously difficult [29,30].

One of the limitations of the present study is the lack of anaerobic Gram-positive cocci in the studied material. The analysis of the anaerobic Gram-positive cocci including *Peptostreptococcus* spp. might be important since the performance of MALDI-TOF MS in identification of gram-positive bacteria is considerably lower than gram-negative bacteria. In addition, MALDI-TOF MS could not identify *P*. *acnes*, *Lactobacillus* species and *C*. *clostridioforme* in the present study. Coltella et. al. (2013) previously reported low performance of MALDI-TOF MS in identification of *P*. *acnes* and *Fusobacterium nucleatum* [11]. The underlying reason remains to be studied. In contrast, identification of the *Bacteroides* groups has shown to be superior with MALDI-TOF MS compared to conventional biochemical methods [31].

In conclusion, MALDI-TOF MS performed equally in all bottle types. However, there was a significant difference in TTD among the four bottles types (p<0.0001), with the shortest median TTD of 18 h in BACTEC Lytic bottles. Furthermore, BACTEC Lytic had significantly higher detection rate than BacT/ALERT FN Plus BC bottles. Further clinical studies are warranted to investigate the performance of anaerobic BC bottles in detection of anaerobic bacteria and identification by direct MALDI-TOF MS.

## Supporting Information

S1 File Data used for the analysis of the performance of the anaerobic blood culture bottles and MALDI-TOF MS. (XLSX)
